# Intraspecific plant–soil feedback in four tropical tree species is inconsistent in a field experiment

**DOI:** 10.1002/ajb2.16331

**Published:** 2024-05-15

**Authors:** Jenalle L. Eck, Lourdes Hernández Hassan, Liza S. Comita

**Affiliations:** ^1^ Yale School of the Environment 195 Prospect St. New Haven 06511 CT USA; ^2^ Smithsonian Tropical Research Institute Luis Clement Ave., Bldg Tupper 401 Ancon Panama Republic of Panama; ^3^ Department of Evolution, Ecology and Organismal Biology The Ohio State University 318 W. 12th Ave., 300 Aronoff Laboratory Columbus 43210 OH USA; ^4^ Department of Botany University of Tartu J. Liivi 2 Tartu 50409 Estonia

**Keywords:** Barro Colorado Island, intraspecific variation, Janzen‐Connell hypothesis, maternal tree, plant genotype, plant population, PSF, seedling survival, soil microbe, tropical forest

## Abstract

**Premise:**

Soil microbes can influence patterns of diversity in plant communities via plant–soil feedbacks. Intraspecific plant–soil feedbacks occur when plant genotype leads to variations in soil microbial composition, resulting in differences in the performance of seedlings growing near their maternal plants versus seedlings growing near nonmaternal conspecific plants. How consistently such intraspecific plant–soil feedbacks occur in natural plant communities is unclear, especially in variable field conditions.

**Methods:**

In an in situ experiment with four native tree species on Barro Colorado Island (BCI), Panama, seedlings of each species were transplanted beneath their maternal tree or another conspecific tree in the BCI forest. Mortality and growth were assessed at the end of the wet season (~4 months post‐transplant) and at the end of the experiment (~7 months post‐transplant).

**Results:**

Differences in seedling performance among field treatments were inconsistent among species and eroded over time. Effects of field environment were detected at the end of the wet season in two of the four species: *Virola surinamensis* seedlings had higher survival beneath their maternal tree than other conspecific trees, while seedling survival of *Ormosia macrocalyx* was higher under other conspecific trees. However, these differences were gone by the end of the experiment.

**Conclusions:**

Our results suggest that intraspecific plant–soil feedbacks may not be consistent in the field for tropical tree species and may have a limited role in determining seedling performance in tropical tree communities. Future studies are needed to elucidate the environmental and genetic factors that determine the incidence and direction of intraspecific plant–soil feedbacks in plant communities.

Plant–soil feedbacks (PSFs) are a key ecological mechanism by which soil‐ and plant‐associated microbes drive species composition and ecosystem functioning in plant communities worldwide (reviewed by Bever, 2003; Bonanomi et al., 2005; Kulmatiski et al., 2008; Crawford et al., 2019). One potential way that PSFs may occur is due to an accumulation of host‐specific microbes, including both mutualists (e.g., mycorrhizal fungi) and antagonists (e.g., microbial pathogens), in the soil around established plants (Bever, 1994; Klironomos, 2002; Packer and Clay, 2003b; reviewed by Ehrenfeld et al., 2005). This accumulation causes the composition of soil microbial communities to vary among co‐occurring plant species (Westover et al., 1997; Osanai et al., 2013; Burns et al., 2015; Fitzpatrick et al., 2018). Consequently, the soil microbial communities near established plants often have differential effects on the survival and/or growth of conspecific versus heterospecific seedlings (see meta‐analyses by Kulmatiski et al., 2008; Crawford et al., 2019). However, the potential role of plant genotype in generating PSFs within species is unclear.

Recent experimental evidence suggests that intraspecific PSFs can occur within populations (Eck et al., 2019; Crawford and Hawkes, 2020) and among populations (Bukowski and Petermann, 2014; Liu et al., 2015; Wagg et al., 2015; Bukowski et al., 2018; Kirchoff et al., 2019) of the same plant species. Within populations, intraspecific PSFs can be characterized by variation in the performance of closely related seedlings (e.g., offspring) versus unrelated seedlings when exposed to the soil microbial communities of conspecific adults. Intraspecific PSFs may occur if microbes are genotype‐specific, i.e., if a given microbial species or community has variable effects on the fitness of different genotypes of a given plant species. This variability may be caused by differences among plant genotypes in their level of resistance to pathogenic species (Alexander et al., 1993; Laine, 2004; reviewed by Gururani et al., 2012) or ability to benefit from mutualistic species (Heath and Tiffin, 2007; Salem et al., 2018; Eck et al., 2022; reviewed by Smith and Goodman, 1999) in their environments. Though genotype specificity in plant–microbial interactions has been confirmed by molecular studies in several crop and model plant species, equivalent studies in wild plants are scarce. Furthermore, evidence is accumulating that microbial community composition can vary among conspecific plants of different genotypes, with corresponding effects on plant fitness or productivity (e.g., Schweitzer et al. 2008; reviewed by terHorst and Zee, 2016). Together, these lines of evidence suggest a potential role for intraspecific PSFs in affecting seedling performance in plant communities.

However, how often intraspecific plant–soil feedbacks occur within species in plant communities and whether they are strong enough to influence seedling performance under field environmental conditions are unclear. Patterns of seedling performance that fit the predictions of negative intraspecific PSFs have been demonstrated in a handful of plant species (Bukowski and Petermann, 2014; Liu et al., 2015; Bukowski et al., 2018; Eck et al., 2019; Kirchoff et al., 2019; Crawford and Hawkes, 2020), but positive intraspecific PSFs (Bukowski and Petermann, 2014) and lack of intraspecific PSFs in some species can occur (Rallo et al., 2023). Furthermore, existing evidence of intraspecific PSFs arises primarily from experiments in controlled conditions (but see Browne and Karubian, 2016; Kirchoff et al., 2019). In a prior shadehouse experiment, we found that seedlings of a tropical tree species [*Virola surinamensis* (Rol. ex Rottb.) Warb.; Myristicaceae] had reduced growth in the soil microbial community from beneath their maternal tree relative to the soil microbial community from beneath nonparent conspecific trees in their population (Eck et al., 2019). Because plant associations with microbes and microbial effects on plant performance can differ between controlled experiments and field conditions (Heinze et al., 2016), it is unclear whether the effects we detected in the shadehouse would translate to patterns of seedling survival in the field. In a field experiment with another tropical tree species, seedlings with rarer genotypes were more likely to survive near conspecific adults than were seedlings with more common genotypes (Browne and Karubian, 2016), suggesting that intraspecific PSFs may also occur in tropical forests.

Despite the importance of PSFs in shaping the composition and diversity of plant communities, it remains unclear how consistently intraspecific PSFs occur among species and over time and whether they are strong enough to be detected in the field. To address this knowledge gap, we conducted a field experiment with four tropical tree species on Barro Colorado Island (BCI), Panama. We asked: Does seedling performance differ beneath maternal conspecific trees versus beneath other conspecific trees under field conditions? The field setting of our experiment allowed us to assess seedling performance and test for evidence of intraspecific PSFs in an ecologically relevant context. With this study, we aimed to better understand how consistently intraspecific PSFs occur within species in a tropical forest community, allowing better predictions regarding the incidence, magnitude, and direction of intraspecific PSFs and aiding our understanding of their consequences for plant diversity.

## MATERIALS AND METHODS

### Study site and species

Our study focused on four canopy tree species on Barro Colorado Island (BCI), Republic of Panama (9°09′N, 79°51′W). The island is a 15.6‐km^2^ moist tropical lowland forest (Croat, 1978), receiving ~2600 mm of rainfall per year, with a distinct dry season from ~January to May (Windsor, 1990). The species represent a range of tropical tree families on BCI: (1) *Lacmellea panamensis* (Woodson) Markgf. (Apocynaceae), (2) *Ormosia macrocalyx* Ducke (Fabaceae), (3) *Tetragastris panamensis* (Engl.) Kuntze (Burseraceae), and (4) *V. surinamensis* (Myristicaceae). *Ormosia macrocalyx*, *T. panamensis*, and *V. surinamensis* are native to much of Central America and northern Amazonia, while *L. panamensis* has a more limited distribution spanning Panama, Costa Rica, and Belize (Croat, 1978). On BCI, the species vary in relative abundance: *T. panamensis* and *V. surinamensis* are common, *L. panamensis* is occasional, while *O. macrocalyx* is rare (Croat, 1978). Seedlings of each species are shade tolerant (Howe, 1990; Gilbert et al., 2006; Myers and Kitajima, 2007; Krause et al., 2012) but vary in drought sensitivity: *L. panamensis* and *T. panamensis* are drought tolerant; *V. surinamensis* is drought sensitive (Kursar et al., 2009; drought tolerance for *O. macrocalyx* is unknown). Seeds of each species are medium‐ or large‐sized (~1–2 cm) and animal‐dispersed, with seed production peaking during March in *T. panamensis* and *L. panamensis*, June in *V. surinamensis*, and September–November and April in *O. macrocalyx* (Croat, 1978; Zimmerman et al., 2007; S. J. Wright, Smithsonian Tropical Research Institute, personal communication). *Virola surinamensis* and *T. panamensis* are dioecious, while *L. panamensis* and *O. macrocalyx* are hermaphroditic (Croat, 1978). These species were chosen because their seeds were available at the time of collection and germinated in sufficient quantities in the shadehouse.

### Field experiment

To test whether seedling performance differed beneath maternal conspecific trees compared to beneath nonmaternal conspecific trees, we conducted a field experiment in the BCI forest. We collected seeds from beneath the canopy of six fruiting *V. surinamensis*, three fruiting *T. panamensis*, seven fruiting *L. panamensis*, and three fruiting *O. macrocalyx* on BCI during late June–early August 2015. We assigned the tree that a seed was collected beneath as the seed's putative parent. Seeds from each parental source were surface‐sterilized (10% v/v bleach for 1 min, rinse, 70% v/v ethanol for 30 s, rinse) and air‐dried. *Ormosia macrocalyx* seeds were scarified and submerged in water for 24 h before planting to encourage germination (Sautu et al., 2007). All seeds were germinated in a shadehouse in autoclaved BCI soil (collected from the forest edge near the shadehouse) under two layers of 80% shadecloth. One month after germination, 145 *V. surinamensis* seedlings, 130 *O. macrocalyx* seedlings, 68 *L. panamensis* seedlings, and 30 *T. panamensis* seedlings were selected for inclusion in the experiment (for a total of 373 experimental seedlings). The number of seedlings per species and seed source reflected availability of seeds collected and healthy seedlings available at the time of transplantation (see Table 1 for an overview of the experimental design).

**Table 1:** Overview of the field experimental design. Seedlings of four tropical tree species (*Virola surinamensis*, *Ormosia macrocalyx*, *Lacmellea panamensis*, and *Tetragastris panamensis*) were grown near their maternal trees or near nonmaternal conspecific trees in a field experiment on Barro Colorado Island (Panama). Included for each species are the number of maternal seed sources (which also served as maternal conspecific field sites for those experimental seedlings that were their offspring), the number of potential nonmaternal conspecific field sites (including non‐offspring experimental seedlings transplanted beneath all seed sources, plus beneath additional nonmaternal conspecific field sites for *O. macrocalyx* and *T. panamensis*), the range in the number of plots per field site, the range in the number of seedlings per plot, the number of seedlings planted in maternal sites, the number of seedlings planted in nonmaternal conspecific field sites, and the total number of experimental seedlings. Variation in variables among species reflects variation in seed and/or seedling availability at the beginning of the experiment. The total number of plots in the experiment is reported rather than the range of plots per site for all species combined.

Species	Seed sources/maternal field sites	Non‐maternal conspecific field sites	Plots per site	Seedlings per plot	Seedlings in maternal sites	Seedlings in nonmaternal conspecific Sites	All seedlings
*V. surinamensis*	6	6	4–6	4–5	65	80	145
*O. macrocalyx*	3	5	5–7	4–5	45	85	130
*L. panamensis*	7	7	1–4	3–5	34	34	68
*T. panamensis*	3	4	2–3	2–3	14	16	30
All species	19	22	90	–	158	215	373

**Figure 1:**
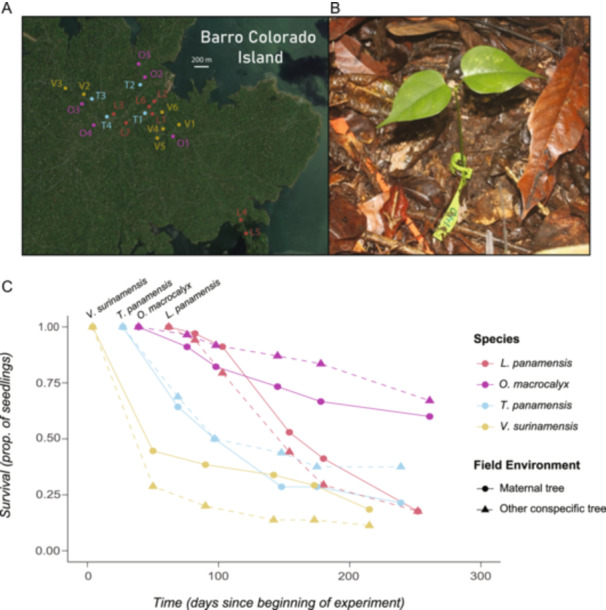
Seedling survival decreased over time in a field experiment on Barro Colorado Island (BCI), Panama. (A) Reproductive trees of four tropical species were used as seed sources and/or seedling transplant sites. Approximate locations of trees of *Virola surinamensis* (V1–V6), *Lacmellea panamensis* (L1–L7), *Ormosia macrocalyx* (O1–O5), and *Tetragastris panamensis* (T1–T4) are marked. (B) In the field experiment, we transplanted seedlings of each species beneath their maternal tree or beneath another conspecific tree in the BCI forest. The photograph shows a stem‐tagged *O. macrocalyx* experimental seedling in a field plot. (C) Proportion of surviving seedlings declined over time in all species and treatments during the field experiment.

Seedlings of each species were randomly assigned to one of two treatments: maternal field environment or nonmaternal conspecific field environment. Seedlings in the first group were transplanted beneath their own maternal tree in the field. Seedlings in the second group were randomly assigned to be transplanted beneath one of the other seed source trees of their same species (other than their maternal tree). Thus, the experimental treatment signified the putative relationship between an experimental seedling and the conspecific tree it was transplanted beneath during the experiment. Both putative offspring and non‐offspring conspecific seedlings were transplanted beneath each maternal seed source. In the hermaphroditic species, nonmaternal conspecific trees cannot be ruled out as potential pollen donors to the seedlings. To limit this possibility, in the dioecious species, only female trees were included in the experiment. To increase the number of nonmaternal conspecific field environments in *T. panamensis* and *O. macrocalyx*, we selected one additional fruiting *T. panamensis* and two additional fruiting *O. macrocalyx* as seedling transplant sites. All focal trees in the experiment were located by exploring a ~5.5‐km^2^ area of BCI with the aid of a mapped 25‐ha plot (provided by S. J. Wright, Smithsonian Tropical Research Institute, personal communication). Focal trees were located ~100 m to ~2 km apart (see Figure 1A for map). All met or exceeded the minimum reproductive diameter for their species (Croat, 1978; Wright et al., 2005).

The experiment was set up one species at a time during late August–October 2015. All experimental seedlings were transplanted at ~1 mo of age into their field treatments. Each seedling was randomly transplanted into one of several seedling plots (1 m^2^) beneath the canopy of their assigned conspecific tree. The number of focal plots per tree (1–7 plots) and the density of experimental seedlings within each plot was determined by seedling availability: Species with more experimental seedlings available had more plots per focal tree and more seedlings per plot (Table 1). Small ranges in the number of conspecific seedlings per plot (2–5 seedlings) were used to minimize the potential impact of conspecific seedling neighbors on seedling survival or survival. Plot locations were randomized with respect to direction and distance from the base of the focal tree (1–4 m). Each seedling was transplanted into a randomly selected 25‐cm^2^ position within a plot. Seedlings roots were not rinsed before transplant.

At the time of transplant, each seedling was stem‐tagged with a unique identification number (see Figure 1B for an example photograph). Stem height, the number of leaves, and the length and width of each leaf were also measured for each seedling at the time of transplant. To estimate initial oven‐dried biomass for each experimental seedling, we used species‐specific allometric equations. We generated these equations from models built using measurements of the stem height, leaf area (measured with a leaf area scanner), and total oven‐dried biomass of a randomly harvested sample of potential experimental seedlings of each species at the beginning of the experiment (*V. surinamensis*: *F*_2,47_ = 496.5, *P* < 0.001, *R* ^2^ = 0.95; *O. macrocalyx*: *F*_2,37_ = 154.7, *P* < 0.001, *R* ^2^ = 0.89; *L. panamensis*: *F*_2,26_ = 183.7, *P* < 0.001, *R* ^2^ = 0.93; *T. panamensis*: F_2,7_ = 19.7, *P* = 0.001, *R* ^2^ = 0.81). All field experimental seedlings were censused every 1–2 mo, during which time survival was recorded, and the stem height and number of leaves were measured for all surviving seedlings. We also recorded instances of seedling stem breakage or uprooting (which we refer to as clipping), likely caused by mammalian herbivores (e.g., agoutis). Seedlings were not watered or enclosed during the field experiment and received only ambient rainfall. Seedlings that were not found during a census (with or without their tags being found) were recorded as dead. After ~7 mo, all surviving seedlings were harvested (*N* = 21, *V. surinamensis* seedlings; *N* = 84, *O. macrocalyx* seedlings; *N* = 12, *L. panamensis* seedlings, and *N* = 9, *T. panamensis* seedlings). Seedlings were harvested one species at a time between late March and early May 2016. Total oven‐dried biomass (including aboveground and belowground portions), stem height, and total leaf area (measured with a scanner) were measured at harvest for each surviving seedling. The final months of the experiments overlapped with a severe dry season due to the strong 2015–2016 El Niño event: signs of wilting were observed in some seedlings (particularly *V. surinamensis*).

### Statistical analyses

To test whether seedling performance in the field experiment differed in maternal conspecific field environments relative to nonmaternal conspecific field environments, we built a series of mixed‐effects models. Because we expected pathogen effects to be stronger in the wet season and because the experiment extended into an unusually severe dry season, we analyzed seedling performance at two time points during the experiment: (1) shortly after the end of the wet season (early‐mid January, ~4 mo into the experiment), to exclude dry season effects, and (2) at the end of the 7‐mo experiment (which coincided with the dry season). Seedling performance at these time points was analyzed using data on seedling survival and seedling relative growth rate (RGR, which was based on measurements of seedling stem height and leaf number during the experiment, or biomass at the end of the experiment).

First, we constructed models examining seedling survival at each time point using data from all four species combined. Seedling survival was modelled as a binary response variable using generalized linear mixed‐models with binomial errors. Field environment (i.e., maternal conspecific or nonmaternal conspecific) and species were included as fixed effects in each model. Because we expected that patterns of seedling performance in the field environments could vary among species, we also included an interaction between field environment and species as a fixed effect in these models. Other fixed effects in these models included estimated initial seedling biomass and seedling clipping (i.e., whether the seedling had experienced clipping up until the time of analysis). The identity of the maternal seed source tree, the identity of the conspecific tree under which the seedling was transplanted (i.e., soil source), and field plot were included as random effects in each model. Initial seedling biomass did not vary significantly among field environments in any focal species.

To look more deeply at patterns of survival in each species, we then built separate survival models for each of the four species in the experiment during each season. The list of fixed and random effects in these models differed from the model described above in the following ways: (1) species and its interaction term with field environment were necessarily excluded; (2) due to a limited number of seed sources in *O. macrocalyx* and *T. panamensis*, seed source was included in these species’ models as a fixed effect rather than a random effect; and (3) for the same reason, soil source was included as a fixed effect rather than a random effect in the *T. panamensis* models. In addition, clipped seedlings and the term denoting clipping were removed from the *O. macrocalyx* and *T. panamensis* models, because clipping was infrequent in these species (4.6% of seedlings in *O. macrocalyx* and 6.6% in *T. panamensis*) and including the term prevented convergence in some models. In species where clipping was more frequent, i.e., *V. surinamensis* (31.7% of seedlings) and *L. panamensis* (16.2% of seedlings), we built two versions of each model: The first excluded clipped seedlings from the model, while the other retained clipped seedlings but included clipping as a fixed effect. Because including clipped seedlings did not affect model results in *V. surinamensis* and *L. panamensis*, we present the results of the models that include clipped seedlings to utilize data on all available seedlings in these species.

We also tested for differences in seedling RGRs between the field environments during the experiment. Seedling RGRs were calculated based on field measurements of stem height and counts of leaves for each living seedling during each census interval as

$$\text{RGR}=\frac{\ln(S_{2})-\ln(S_{1})}{t_{2}-t_{1}},$$

where *S*_1_ = size at time one (i.e., estimated dry biomass in grams), *S*_2_ = size at time two, *t*_1_ = time one (in days elapsed since the seedling was transplanted), and *t*_2_ = time two.

This resulted in a total of 959 RGR observations from 260 seedling individuals across five census intervals. The RGR data were analyzed at the end of the wet season and at the end of the experiment using linear mixed‐effects models that included data from all four species. The RGR models included the same set of fixed and random effects specified for the all‐species survival model. In addition, the RGR models included seedling ID (to account for repeated measurements of seedlings over time) and census interval as random effects. Because the number of surviving seedlings was small in some species (Figure 1C), we did not construct separate RGR models for each species. All statistical analyses in our study were conducted in the R statistical environment version 4.3.1 (R Core Team, 2023). Linear mixed‐effects models in our study were constructed using the R package lme4 (Bates et al., 2015). *P* values for mixed‐effect model predictors were obtained using R packages lmerTest (Kuznetsova et al., 2016) and car (Fox and Weisberg, 2019). Long‐format data for the RGR analyses were obtained using R package tidyr (Wickham et al., 2023). Plots were generated using R packages ggplot2 (Wickham, 2016) and interactions (Long, 2019).

## RESULTS

### Seedling survival in the field experiment

**Figure 2:**
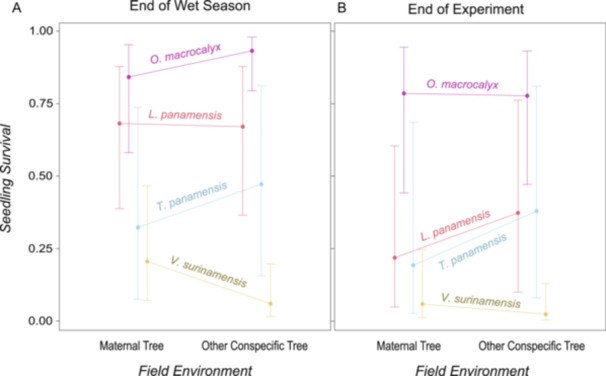
Patterns of seedling survival near maternal conspecific trees versus nonmaternal conspecific trees varied among four species at the end of the wet season but not at the end of the experiment on Barro Colorado Island, Panama. (A) Field environment (maternal conspecific tree versus nonmaternal conspecific tree) and species interacted to jointly determine seedling survival in four focal tropical tree species at the end of the wet season (Appendix S1: Table S1; *P* = 0.03, *N* = 373 seedlings). (B) By the end of the experiment, patterns of seedling survival in the field treatments were similar among species (though species‐level differences in survival remained; Appendix S1: Table S1; *P* = 0.32, *N* = 373 seedlings). In each panel, the mean estimated survival probability predicted by a generalized linear mixed‐effects model is plotted for each species and treatment group. Error bars on the estimates represent a 95% confidence interval.

**Figure 3:**
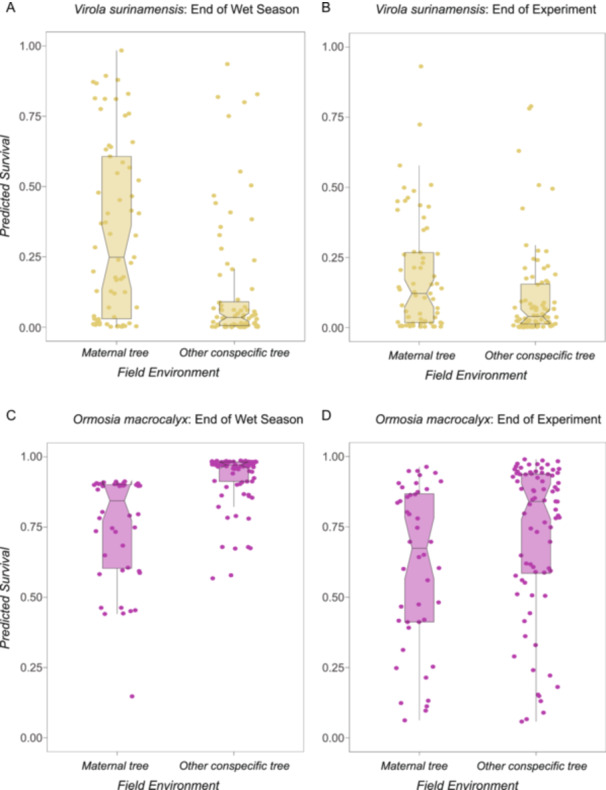
Opposite patterns of seedling survival near maternal conspecific trees versus nonmaternal conspecific trees were observed in *V. surinamensis* and *O. macrocalyx* at the end of the wet season on Barro Colorado Island, Panama, but these differences eroded over time. (A) Survival of *Virola surinamensis* seedlings at the end of the wet season was higher when grown beneath their maternal tree than beneath a nonparent conspecific female tree (Appendix S1: Table S2; *P* = 0.02, *N* = 145 seedlings). (B) By the end of the experiment, differences in *V. surinamensis* seedling survival were similar among conspecific field environments (Appendix S1: Table S2; *P* = 0.25, *N* = 145 seedlings). (C) In contrast, survival of *Ormosia macrocalyx* seedlings at the end of the wet season was lower beneath the maternal tree than beneath other conspecific trees (Appendix S1: Table S3; *P* = 0.05, *N* = 124 seedlings). (D) *Ormosia macrocalyx* seedling survival was similar among treatments by the end of the experiment (Appendix S1: Table S3; *P* = 0.92, *N* = 124 seedlings). In each panel, dots represent the predicted value for each seedling, box belts show the predicted median values, box notches represent a 95% confidence interval for comparing predicted medians, box hinges correspond to the first and third quartiles, and box whiskers extend to the largest and smallest value no farther than 1.5× the interquartile range from the hinges.

Patterns of seedling survival in maternal field environments versus nonmaternal conspecific field environments varied among species at the end of the wet season (Figure 2A; Appendix S1: Table S1; *P* = 0.03, *N* = 373 seedlings), but were similar among species by the end of the experiment (Figure 2B; Appendix S1: Table S1; *P* = 0.32, *N* = 373 seedlings). At the end of the wet season, seedlings of *V. surinamensis* had higher survival when growing beneath their maternal tree versus beneath another female conspecific tree (Figure 3A; Appendix S1: Table S2; *P* = 0.02, *N* = 145 seedlings). In contrast, seedlings of *O. macrocalyx* had lower survival at the end of the wet season when growing beneath their maternal tree (Figure 3C; Appendix S1: Table S3; *P* < 0.05, *N* = 124 seedlings). Differences in seedling survival between field environments in these species disappeared during the dry season (Figures 3B, D; Appendix S1: Tables S2, S3). Seedling survival was similar between field environments in *L. panamensis* (Appendix S1: Table S4) and *T. panamensis* (Appendix S1: Table S5) at the end of the wet season and the end of the experiment. Rates of seedling survival also varied among species (Appendix S1: Table S1; *P* < 0.01, *N* = 373 seedlings). Seedlings with higher initial biomass were more likely to survive (Appendix S1: Table S1; *P* < 0.01, *N* = 373 seedlings), while clipped seedlings were less likely to survive (Appendix S1: Table S1; *P* = 0.02, *N* = 373 seedlings).

### Seedling growth in the field experiment

**Figure 4:**
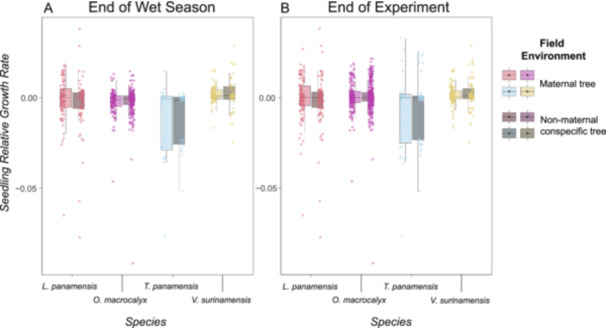
Seedling relative growth rate varied among species but was similar among field environments during the experiment on Barro Colorado Island, Panama. Relative growth rates of seedlings of four tropical tree species were similar among field environments (maternal tree versus another conspecific tree) (A) at the end of the wet season (Appendix S1: Table S6; *P* = 0.87, *N* = 668 observations) and (B) at the end of the experiment (Appendix S1: Table S6; *P* = 0.55, *N* = 959 observations). In each panel, dots represent the predicted value for each seedling, box belts show the predicted median values, box hinges correspond to the first and third quartiles, and box whiskers extend to the largest and smallest value no farther than 1.5× the interquartile range from the hinges.

Seedling RGRs did not vary between field environments and did not change over time throughout the experiment (Figure 4A, B; Appendix S1: Table S6). Relative growth rates varied among species throughout the experiment (Appendix S1: Table S6; p < 0.01, *n* = 959 observations). In addition, seedlings with higher initial biomass had higher RGRs during the experiment (Appendix S1: Table S6; *P* < 0.01, *N* = 959 observations), while clipping reduced seedling RGRs (Appendix S1: Table S6; *P* < 0.01, *N* = 959 observations).

## DISCUSSION

In our in situ field experiment with four tropical tree species in Panama, we did not find evidence that intraspecific plant–soil feedbacks play a significant role in shaping seedling mortality in the field. Though interspecific PSFs are well documented (e.g., Crawford et al., 2019), relatively few studies have tested for intraspecific PSFs, especially under ecologically relevant field conditions. In our experiment, patterns of seedling mortality indicative of intraspecific PSFs were not maintained over time and were detected (temporarily) only in two of the four focal species. At the end of the wet season, seedling survival was higher in maternal conspecific field environments relative to nonmaternal conspecific field environments for *V. surinamensis* (consistent with positive intraspecific PSF), but lower for *O. macrocalyx* (consistent with negative intraspecific PSF). In both species, these patterns were no longer present by the end of the experiment. Thus, our field experiment suggests that intraspecific PSFs may play a limited role in determining seedling performance in tropical tree communities.

In a prior shadehouse experiment with the same population of *V. surinamensis* on BCI, we found that seedling growth and colonization by arbuscular mycorrhizal fungi were reduced in the soil microbial community from beneath maternal trees relative to those in the soil microbial community from beneath other female conspecific trees (Eck et al., 2019). Thus, we found patterns of seedling growth that were consistent with negative intraspecific PSF in the shadehouse but did not find similar patterns consistent with intraspecific PSF in the field in this species. In the field, patterns of seedling survival in *V. surinamensis* matched those expected due to positive intraspecific PSF at the end of the wet season, but these effects were not maintained over time, and we found no effect of intraspecific PSF overall. In general, differences within species in the direction of intraspecific PSFs for growth versus survival could indicate a growth‐defense trade‐off for seedlings in the soil microbial communities near their maternal tree. This trade‐off could occur, for example, if the accumulation of genotype‐specific pathogens in the soil near adult trees activates seedling defenses, reducing the resources available for seedling growth but increasing the seedlings’ chance of survival. In our field experiment, seedling mortality rates were high but growth rates were low, indicating the challenges of measuring these metrics in field experiments in tropical forests.

Conflicting findings between these studies may reflect differences in conditions in the shadehouse versus field. Soil microbial effects that are easily isolated in controlled conditions in the shadehouse may be modified by other ecological variables in the field. In our prior shadehouse experiment, seedlings were grown in a common sterilized soil medium that was inoculated with a small volume of field soil. Thus, the negative intraspecific plant–soil feedback we observed in that study could be attributed entirely to soil microbes. In contrast, in the present study, seedling performance may have been affected not only by conditioned soil microbes, but by differences in soil nutrients or biochemistry, microclimate, herbivore composition, or other unmeasured factors caused by the adult trees. In addition, it is possible that other environmental factors such as topography, light levels, soil type or moisture, and vegetation composition varied near the conspecific trees. Environmental variables such as soil conditions (Wei et al., 2018) and plant litter (Veen et al., 2019) have been shown to play a role in determining PSFs (reviewed by van der Putten et al., 2016; De Long et al., 2018), and environmental factors other than soil microbes could have affected seedling survival (Johnson et al., 2017). In addition, the cause of mortality was not usually known for the seedlings in our study. Ultimately, it is unsurprising for different patterns to arise in shadehouse versus field studies because several factors can potentially modify or override the effects of soil microbes in the field; thus, one may not necessarily predict the other (Beckman et al., 2022).

Variation in the incidence, direction, and magnitude of interspecific plant–soil feedbacks among species within plant communities has often been documented (e.g., Mangan et al., 2010; Reinhart, 2012; Bennett et al., 2017). In our study, *V. surinamensis* and *O. macrocalyx* briefly demonstrated opposite patterns of intraspecific PSF for seedling survival. Variation in intraspecific PSFs among plant species might be expected due to variation in species’ genetic resources, growth and defensive traits, soil microbial associations, and life history. However, characteristics of interspecific PSF might not correlate with characteristics of intraspecific PSF within plant species. In another previous shadehouse experiment with three of our focal species on BCI, *V. surinamensis* and *L. panamensis* exhibited negative interspecific PSFs, while *T. panamensis* did not exhibit interspecific PSF (Mangan et al., 2010). In our study, these species did not exhibit intraspecific PSF by the end of the experiment.

Simulation studies have shown that negative intraspecific PSF can help maintain species diversity (albeit, less strongly than negative interspecific PSF) and is most effective at maintaining species richness when genetic diversity is relatively low (Eck et al., 2019). Genetic relatedness between seedlings and conspecific adult plants has been shown to predict seedling survival in the soil near those adults: soil microbial communities promoted the survival of seedlings from more genetically distant populations (Liu et al., 2015). Variation in seedling performance in conspecific field soils has also been demonstrated to favor the survival of seedlings of locally rare genotypes (Browne and Karubian, 2016) and promote genetic diversity within plant populations (Browne and Karubian, 2018). Together, these studies suggest a role for plant genotype in determining patterns of seedling performance and diversity in the field.

Our findings also indicate that patterns of intraspecific plant–soil feedback could vary over time, potentially due to several factors. Interspecific PSFs are also thought to vary temporally (Packer and Clay, 2003a; Hawkes et al., 2013; reviewed by Kardol et al., 2013; Chung, 2023), to occur more often in stable relative to variable environments (Duell et al., 2019), and to be more negative in temporally stable patches (Chung et al., 2019). Changing environmental conditions in the field could drive changes in intraspecific PSF. In our experiment, patterns of intraspecific PSF emerged during the wet season, but disappeared by the end of the following dry season. Our experiment coincided with the 2015–2016 El Niño event that resulted in a severe dry season, which was linked to elevated seedling mortality in our study region (Browne et al., 2021). Drought has been shown to influence PSFs (Kaisermann et al., 2017; Fry et al., 2018; reviewed by Hassan et al., 2021; de Vries et al., 2023). In addition, intraspecific PSF effects could be strongest during certain seedling developmental phases (reviewed by Kardol et al., 2013). Our study would miss any effects that might impact seed survival, germination, very early seedling mortality, or later‐stage seedling performance. Additional studies are needed to disentangle the effects of environmental variation versus developmental age on changes in intraspecific PSFs.

Plant–soil feedbacks are one mechanism by which soil microbes may drive patterns of species diversity in plant communities, but how consistently PSFs occur among genotypes within plant species is unclear, especially under variable field conditions. Intraspecific variability in biotic interactions is often overlooked in community ecology but can greatly affect ecological patterns (reviewed by Freckleton and Lewis, 2006; Bolnick et al., 2011). To date, few studies have tested for evidence of intraspecific PSFs in the field, where seedlings are exposed to their full set of natural enemies (see also Browne and Karubian, 2016; Kirchoff et al., 2019). Intraspecific variation in seedling performance near conspecific adults, if it occurs, could be consequential because of its potential to influence species and/or genetic diversity in plant communities (Stump and Chesson, 2015; Browne and Karubian, 2018; Eck et al., 2019). Future studies are needed to elucidate the environmental, genetic, or trait‐based factors that determine the incidence and direction of intraspecific plant–soil feedbacks in plant communities.

## CONCLUSIONS

Understanding the causes and consequences of variability in plant–microbe interactions and plant–soil feedbacks is a key challenge in plant biology. We demonstrated via a field experiment with four tropical tree species in Panama that the patterns of seedling performance indicative of intraspecific plant–soil feedback do not occur consistently among species and erode over time. Thus, our study suggests a limited role of intraspecific plant–soil feedback in determining patterns of seedling performance in tropical tree communities.

## AUTHOR CONTRIBUTIONS

J.L.E. and L.S.C. designed the experiments. J.L.E. and L.H.H. set up the experiments and collected the data. J.L.E. analyzed the data with help from L.S.C. J.L.E. wrote the first draft of the manuscript. All authors contributed to and approved the final version of the manuscript.

## Supporting information


**Table S1**. Effect of maternal conspecific versus other conspecific field environments on seedling survival in four species.
**Table S2**. Effect of maternal conspecific versus nonparent female conspecific field environments on *Virola surinamensis* seedling survival.
**Table S3**. Effect of maternal conspecific versus other conspecific field environments on *Ormosia macrocalyx* seedling survival.
**Table S4**. Effect of maternal conspecific versus other conspecific field environments on *Lacmellea panamensis* seedling survival.
**Table S5**. Effect of maternal conspecific versus nonparent female conspecific field environments on *Tetragastris panamensis* seedling survival.
**Table S6**. Effect of maternal conspecific versus other conspecific field environments on seedling relative growth rates in four species.

## Data Availability

Data and scripts supporting this study are available from the Dryad Digital Repository: https://doi.org/10.5061/dryad.0cfxpnw8p (Eck et al. 2024).
